# Towards Early Diagnosis and Screening of Alzheimer’s Disease Using Frequency Locked Whispering Gallery Mode Microtoroid Biosensors

**DOI:** 10.21203/rs.3.rs-4355995/v1

**Published:** 2024-05-17

**Authors:** Adley Gin, Phuong-Diem Nguyen, Geidy Serrano, Gene Alexander, Judith Su

**Affiliations:** The University of Arizona; The University of Arizona; Barrow Neurological Institute; The University of Arizona; The University of Arizona

## Abstract

Alzheimer’s disease (AD) is a progressive form of dementia affecting almost 55 million people worldwide. It is characterized by the abnormal deposition of amyloid plaques and neurofibrillary tangles within the brain, leading to a pathological cascade of neuron degeneration and death as well as memory loss and cognitive decline. Amyloid beta (Aβ) is an AD biomarker present in cerebrospinal fluid and blood serum and correlates with the presence of amyloid plaques and tau tangles in the brain. Measuring the levels of Aβ can help with early diagnosis of AD, which is key for studying novel AD drugs and delaying the symptoms of dementia. However, this goal is difficult to achieve due to the low levels of AD biomarkers in biofluids. Here we demonstrate for the first time the use of FLOWER (frequency locked optical whispering evanescent resonator) for quantifying the levels of post-mortem cerebrospinal fluid (CSF) Aβ42 in clinicopathologically classified control, mild cognitive impairment (MCI), and AD participants. FLOWER is capable of measuring CSF Aβ42 (area under curve, AUC = 0.92) with higher diagnostic performance than standard ELISA (AUC = 0.82) and was also able to distinguish between control and MCI samples. Our results demonstrate the capability of FLOWER for screening CSF samples for early diagnosis of Alzheimer’s pathology.

## INTRODUCTION

As of 2023, Alzheimer’s disease (AD) is the most common cause of age-related dementia affecting an estimated 6.7 million Americans that are age 65 and older^1^. By the year 2030, that number is projected to increase by 26% as the elderly demographic of the U.S. population grows^2^. In 2019, AD was the 6th leading cause of death in the U.S., and for people above age 70, 61% of those with AD are expected to die before 80 compared to the 30% of people without the disease^3^. AD is a progressive disorder beginning with biological changes within the brain of individuals 20 years or more before they exhibit symptoms of cognitive decline^4–6^. These biological changes include the abnormal accumulation of amyloid plaques and formation of neurofibrillary tangles within the brain, which are thought to cause a cascading effect of neuron degeneration leading to declining brain function and memory loss^7,8^. Some of these individuals go on to develop mild cognitive impairment (MCI) before advancing to stages of more severe dementia due to AD. Currently, there are no FDA approved treatments that can prevent or cure AD, but research into therapeutic drugs for removing amyloid plaques in the brain and possibly slowing the progress of dementia in some MCI and early-stage AD patients is ongoing.^9^ Therefore, early diagnosis of AD is crucial for efforts focused on significantly delaying the symptoms of dementia, improving quality of life, and reducing the long-term cost of care for individuals diagnosed with AD.

Amyloid beta (Aβ) is a biomarker of interest for early diagnosis of AD due to its correlation with amyloid plaque and neurofibrillary tangle deposition in the brain^10–12^. Aβ is a 36–43 amino acid residue peptide generated normally throughout life by the amyloid precursor protein (APP), although the normal function of both APP and Aβ is not entirely clear. The most common form of Aβ produced by APP ends at amino acid position 40 (Aβ40, ~ 80–90%), whereas Aβ ending at position 42 (Aβ42, ~ 5–10%) has greater neurotoxicity and is thought to have a preferential role in the formation of AD-related amyloid plaques.^13^ AD amyloid plaques are formed from aggregated extracellular fibrils of Aβ and have been associated with cognitive impairment and dementia. Aβ peptide makes up ~ 70–75% of the protein content present in amyloid plaques.^14^ On the other hand, AD-related intracellular neurofibrillary tangles are composed of hyperphosphorylated tau, an abnormal microtubule associated protein (MAP) that disrupts the structure and assembly of microtubles in the brain^15^. Physicians can measure brain amyloid and tau pathology *in-vivo* with positron emission topography (PET), which allows for the spatial and longitudinal study of how AD progresses over time^16^. Assays for biofluids, such as CSF and plasma, have shown great promise for measuring amyloid and tau as lower cost *in-vivo* biomarkers for early AD detection.^17^ A gold-standard for *in-vitro* measurements of CSF amyloid beta has been the ELISA (enzyme linked immunosorbent sandwich assay).^18^ Ultrasensitive assays for measuring amyloid beta and tau AD biomarkers include electrochemiluminescence assay (ECLIA)^19,20^, single molecule array (SIMOA)^21,22^, mass spectrometry^23,24^, among others. These assays have high sensitivity and good diagnostic performance, but typically require costly instrumentation, multi-step incubations, and fluorescent or isotopic labels which further increase cost and complexity. Additionally, when using CSF Aβ for AD diagnosis, some assays have measured both Aβ40 and Aβ42 to obtain the CSF Aβ42/Aβ40 ratio, which gives better sensitivity and specificity than just Aβ42 alone with established methods.^25^ However, this also comes with increased cost and assay time.

To address these issues, we utilized a technique known as FLOWER which is based on optical microcavity technology. In these experiments, we used microtoroid optical microcavities. FLOWER can perform label-free, ultra-sensitive, real-time measurements of biomolecular binding and is capable of single molecule detection.^26–36^ Based on the principles of total internal reflection and constructive interference, confined photons circulate within the microtoroid sensing element. The unique structure of microtoroids results in heightened sensitivity due to the long (on the order of nanoseconds) photon lifetime and that fact that a portion of the evanescent light field extends past the resonator cavity and into the external medium. This evanescent tail enables microtoroids to detect minute changes in the local refractive index near the sensor’s surface, such as during biomolecular binding. Recent work employing FLOWER includes detecting volatile organic compounds,^34,37^ cancer associated biomarkers^31,38^, performance enhancing drug detection^39^, proteins^40^, and drugs for COVID-19^35^, among others.

In this study, FLOWER was used for the first time to measure Aβ 1–42 (Aβ42) in post-mortem cerebrospinal fluid (CSF) from control, MCI, and AD diagnosed participants. We fabricated microtoroid resonators on-chip from thermal oxide silicon wafers and functionalized them with Aβ42 specific antibody. Using the antibody functionalized toroids, CSF Aβ42 levels were quantified *in-vitro* by measuring the resonant wavelength shift due to biomolecular binding between the Aβ42 and a detection antibody. With FLOWER, we measured significant differences in CSF Aβ42 levels between control, MCI, and AD participant samples which is valuable for early diagnosis of AD. Compared to ELISA, the FLOWER assay demonstrated higher diagnostic performance for CSF Aβ42 (FLOWER AUC = 0.92 vs. ELISA AUC = 0.82).

## MATERIALS AND METHODS

### Materials.

APTES ((3-Aminopropyl)triethoxysilane) was purchased from Sigma Aldrich (SKU 440140). Denatured ethanol was purchased from Honeywell (SKU 270741). The silicon substrate with 2 μm oxide layer was purchased from University Wafer (UniversityWafer, Inc.). Purified anti-β-Amyloid, 1–16 Antibody (6E10 clone) was purchased from Biolegend (Cat # 803001). Purified anti-β-Amyloid, 1–42 Antibody (12F4 clone) was purchased from Biolegend (Cat # 805509). HFIP Aβ42 was purchased from Anaspec (Cat # AS-64129–05). Sulfo-NHS (Cat # 24510) and EDC (Cat # 22980) were purchased from ThermoFisher. Invitrogen ultrasensitive, human amyloid-beta 1–42 ELISA kit was purchased from ThermoFisher (Cat # KHB3544).

## Human Subject Characterization and CSF Sample Collection

Postmortem CSF was collected at autopsy from clinically and neuropathologically characterized participants enrolled in the Arizona Study of Aging and Neurodegenerative Disorders and Brain and Body Donation Program^41^. Briefly, most subjects were volunteers recruited from the surrounding communities of Maricopa County, Arizona, especially the Sun Cities. The demographics of the population consisted largely of Caucasian, middle to high income individuals. For each participant, a subspecialist cognitive-behavioral neurologist performed a comprehensive evaluation. A cognitive diagnosis was assigned at a consensus conference attended by neuropsychologists, neurologists, and cognitive neurology subspecialists. All neuropathological examinations were performed by the same neuropathologist, blinded to clinical findings.

Prior to removing the brain, cerebrospinal fluid (CSF) was drawn from the lateral ventricles, using 30 ml disposable syringes fitted with 8 cm long, 18-gauge needles. The CSF was ejected into 15 ml disposable polyethylene tubes for centrifugation. CSF was centrifuged at 5k rpm for 10 minutes and supernatants from the CSF were aliquoted into 0.5 ml polyethylene microcentrifuge tubes and stored frozen at − 80°C.

Amyloid plaque and neurofibrillary tangle density in brain were graded and staged at standard sites in frontal, temporal, parietal and occipital cortex as well as hippocampus and entorhinal cortex, based on the aggregate impression from the 80 μm sections stained with thioflavine S, Campbell-Switzer and Gallyas methods. The total plaque score, considering all types of plaques (cored, neuritic and diffuse) together, was predominantly derived from the Campbell-Switzer stain while the Gallyas and thioflavine S stains were used for estimating neuritic plaque densities. All three stains show neurofibrillary changes and therefore this score was estimated after viewing slides stained with all three. Both total and neuritic plaque densities were rated as none, sparse, moderate and frequent, using the published CERAD templates^42^. Conversion of the descriptive terms to numerical values provides scores of 0–3 for each area, with a maximum score of 15 for all five areas combined. Neurofibrillary tangle abundance and distribution was also graded in these thick sections, again using the CERAD templates for this, while the original Braak protocol^43^ was used for estimating the topographical distribution of neurofibrillary change.

### Mini-Mental State Examination.

The Mini-Mental State Examination (MMSE) is a brief, widely-used screening test for measuring cognitive impairment and dementia. The test score ranges from 0–30, with lower scores indicating poorer performance and cutoff scores for cognitive impairment typically range between 24–26 with varying sensitivity and specificity.^44^

### FLOWER microtoroid biosensor.

**Figure 1:**
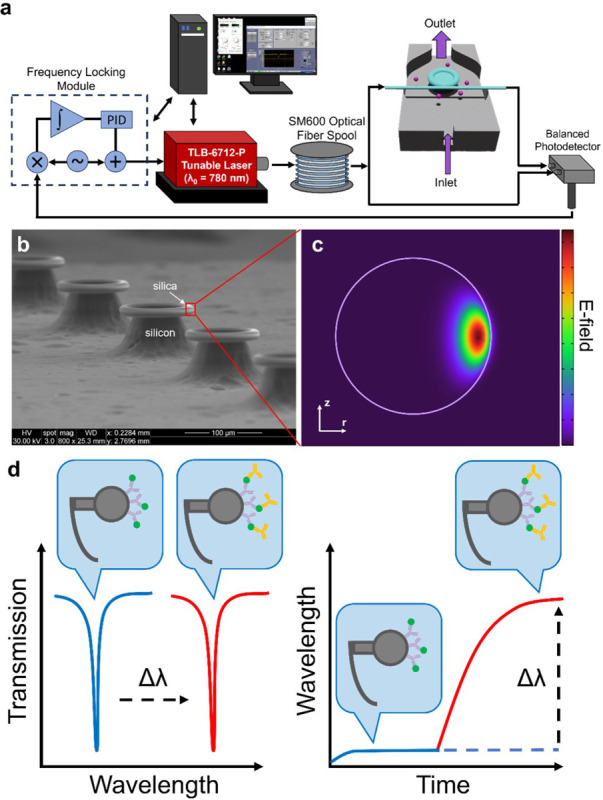
FLOWER experimental setup. (a) Diagram of the components in the FLOWER system and setup of the tapered optical fiber and microtoroid in the custom fluidic chamber. (b) Scanning electron micrograph of a row of silica microtoroids on a silicon substrate. (c) COMSOL simulation of the electric field inside the cross-section of a toroid. Note the evanescent field extending past the toroid’s surface into the surrounding medium. (d) Illustration of an antibody functionalized toroid and the subsequent resonant wavelength shift due to the binding of detection antibody.

The FLOWER biosensing system is illustrated in Fig. 1a, including the major components required for measuring the resonant wavelength shift by frequency-locking a tunable laser (TLB-6712, Newport) to the microtoroid resonator. A narrow linewidth laser with a tuning range from 765 nm to 781 nm was chosen, where the absorption by water is minimal compared to higher infrared wavelengths. Depending on the microtoroid geometry, each microtoroid will support an optical resonance at the resonance condition:

(1) $$2\pi Rn_{eff}=m\lambda$$

where *R* is the major radius of the microtoroid resonator, *n*_*eff*_ is the effective refractive index of the guided mode, *m* is an integer, and *λ* is the free-space wavelength of the laser. Figure 1b shows a scanning electron micrograph of a row of microtoroids on a single chip. The chip is placed in a custom-built fluidic chamber mounted on a 3-axis micrometer and nano positioning piezo stage, which allows for precise coupling between the tapered fiber waveguide and the microtoroid. A 2D axially symmetric COMSOL simulation shows how the optical mode is distributed in a cross section of the microtoroid resonator, along with the evanescent field extending past the surface of the microtoroid and into the surrounding environment (Fig. 1c). Optical resonances appear as sharp dips in the transmission spectrum, and the resonant wavelength shift is measured while the detection antibody is injected into the fluidic chamber (Fig. 1d).

## Microtoroid Fabrication

Silicon wafers with a 2 μm thick thermal oxide layer were purchased from UniversityWafer, Inc. In a cleanroom, a maskless, direct-write photolithography tool (Heidelberg Instruments MLA150) was used to create columns of 150 μm photoresist circles on the wafer. The photoresist pattern acts as a mask during the subsequent buffered oxide wet-etch process, which etches the exposed silica and leaves behind 150 μm silica circles on a silicon substrate. Afterwards, the photoresist mask is washed away with acetone and IPA, and the wafer is cut into smaller “chips” before drying in an oven at 130°C for at least 30 minutes. Next, the chips are dry etched using XeF_2_ (Xactix e2, SPTS) which isotropically etches the exposed silicon substrate to form silica microdisks atop a silicon pillar. A CO_2_ (λ_0_ = 10.5 μm) laser is used to reflow the silica microdisks to form the final microtoroid resonator.

### Microtoroid Surface Functionalization Using APTES.

Prior to the biosensing experiment, microtoroid chips were incubated in a solution of 1% v/v APTES in chloroform for 15 minutes. Next, the chips were washed with denatured ethanol and dried under a nitrogen stream. Afterwards, the chips were incubated overnight in a solution of 0.1 M succinic anhydride in dimethyl formamide. The next day, the chips were washed with denatured ethanol and dried under a nitrogen stream. Next, the chips were incubated in a solution of 100 mM EDC, 100 mM Sulfo-NHS prepared in MES buffer (pH 6) for 15 minutes at room temperature. Afterwards, the chips were washed with 100 mM PBS before incubation in Aβ42 specific antibody.

### Amyloid-beta 1–42 Standard Curve and CSF FLOWER Assay.

To construct a standard curve for Aβ42, the APTES functionalized microtoroids were conjugated with 10 μg/mL anti- Aβ42 (12F4 clone) capture antibody in PBS, which binds to epitopes 36–42 (C-terminal) of the Aβ42 peptide^45^. Dried HFIP Aβ42 (AS-64129–05, Anaspec) peptide film was dissolved in 10 mM NaOH to a stock concentration of 1 mg/mL, and then serially diluted in 100 mM PBS. Next, the chips were incubated in the HFIP treated Aβ42 peptide for 2 hours at room temperature and then washed with HEPES sample buffer (HEPES 25 mM, NaCl 125 mM, BSA 0.1% w/v, EDTA 1 mM, pH 7.5). The chips were kept in HEPES sample buffer on ice until data measurement. The resonant wavelength shift was measured while the anti- Aβ42 detection antibody^46^ (6E10 clone) (1 μg/mL, diluted in HEPES sample buffer) was perfused into the fluidic chamber containing the microtoroid chip. To measure Aβ42 in CSF, the microtoroid chips were functionalized with 10 μg/mL anti- Aβ42 capture antibody (12F4) in PBS. CSF samples were thawed on ice from − 80 degrees C and then centrifuged for 10 minutes at 3000 x g. The chips were incubated in the CSF samples for 2 hours at room temperature before washing with sample buffer. Samples were kept on ice in HEPES sample buffer until the resonant wavelength shift was measured using 1 μg/mL anti- Aβ42 detection antibody (6E10).

### CSF Ultrasensitive Human Aβ42 ELISA.

To compare the diagnostic performance of FLOWER vs. ELISA, we measured CSF Aβ42 using an Invitrogen ultrasensitive human Aβ42 ELISA kit. CSF samples were diluted 1:4 using the included standard diluent buffer before being added to the wells on a 96-well plate and incubated for 3 hours with detector antibody at room temperature. After, the wells were washed 4 times with 1X wash buffer and the HRP conjugated secondary antibody was added and incubated for 30 minutes at room temperature. Next, the wells were washed and Tetramethylbenzidine (TMB) solution was added and incubated for 30 minutes in the dark. Lastly, stop solution was added to each well before reading the plate. The absorbance at 450 nm was measured using a Biotek Synergy HT Microplate Reader.

### FLOWER Experimental Setup and Data Acquisition.

A single-mode optical fiber (SM600, Thorlabs) was tapered using a custom built, motorized pulling stage and a hydrogen torch. After tapering, the fiber remains in the pulling stage and is moved over to the experimental setup and table (See Fig. S1). The functionalized microtoroid chip is affixed with double sided tape in a custom 3D-printed fluidic chamber (internal volume ~ 120 μl) attached to a rod. The rod is mounted onto a 3-axis micrometer and nanopositioning stage (P-611.3 Nanocube, PI) to allow for precise coupling between the microtoroid and the tapered fiber. A glass coverslip is cut to size and placed on top of the fluidic chamber to contain the fluid, chip, and tapered fiber. A 100 μm diameter perfusion pencil tip (AutoMate Scientific) is inserted into the fluidic chamber to allow for delivery of the samples to the toroid via an 8-channel pressurized perfusion system (AutoMate Scientific) and electric rotary valve system (ASP-ERV-O1.2–08, Aurora Pro Scientific). Each chip contains ~ 6–8 toroids, which are checked for high-Q resonances (Q > 10^5^) by evanescently coupling the tapered fiber and measuring the transmission spectrum over the laser’s tuning range.

The shift in the microtoroid resonance frequency is measured using the top-of-fringe locking function on a Toptica Digilock 110. The photodetector’s (Nirvana 2007, Newport) signal output was connected to the Digilock input, and the Digilock analog voltage output was connected to the tunable laser’s frequency modulation input and an analog voltage data acquisition card (DAQ) (PCI-4461, National Instruments). The Digilock modulates the laser’s frequency with a 2 kHz sine wave to generate an error signal. Any shift in the microtoroid resonance frequency is compensated for by the Digilock sending a voltage signal to both the laser and the DAQ card (PCI-4461, National Instruments).

### Wavelength Shift Curve Fitting.

The resonant wavelength shift data was fitted using a one-phase association function describing the binding kinetics between a receptor and its ligand^47^

(2) $$y=Y_{\text{max}}\left(1-{\text{exp}}^{-kx}\right)$$

where *Y*_*max*_ is the projected maximum shift at infinite time with units of femtometer and *k* is a rate constant with units of s^− 1^.

The maximum wavelength shifts obtained from Eq. 2 were used to construct calibration curves for Aβ42 using a 5-parameter logistic fit^48^:

(3) $$y=A_{1}+\frac{A_{2}-A_{1}}{{\left(1+10^{\left(Logx_{0}-x\right)p}\right)}^{s}}$$

where *A*_1_ is the bottom asymptote, *A*_2_ is the top asymptote, *Logx*_0_ is the center, *p* is the hill slope, and *s* is the symmetry factor.

### Statistical Analysis.

We performed Spearman rho correlation analysis between CSF Aβ42, MMSE score, total plaque density score, and total tangle density score. The total CSF Aβ42 measurements between control, MCI, and AD groups were compared using t-tests with a P < 0.05 significance level.

## RESULTS AND DISCUSSION

### Calibration Curves.

**Figure 2:**
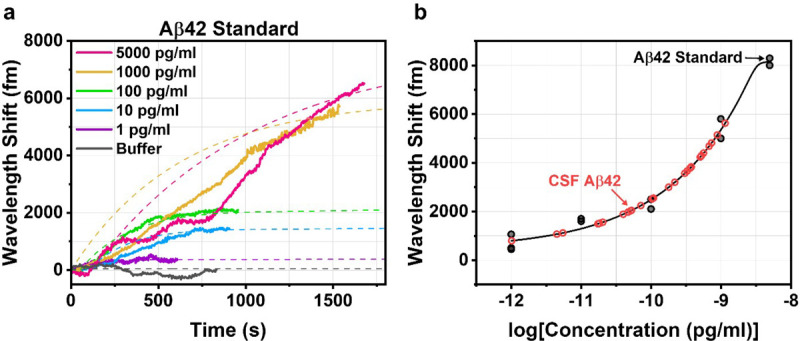
Standard curves for Aβ42 FLOWER assays. (a) Wavelength shift over time as Aβ42 standard binds to the toroid from a representative experiment. (b) Standard curve constructed from Aβ42 binding in repeated experiments. Red circles correspond to post-mortem CSF Aβ42 samples.

To quantify the levels of CSF Aβ42, a standard curve was constructed from known concentrations of Aβ42 standard (See Methods). Prior to measurement, each toroid chip was placed into the fluidic chamber and coupled with the tapered fiber to find a high Q resonance (Q > 10^5^). During this time, sample buffer was constantly perfused into the chamber until a steady state was reached. At the start of the sample measurement, the laser was frequency locked to the toroid and the resonant wavelength shift was recorded while the detection antibody (1 μg/mL) was constantly perfused into the chamber (Fig. 2a). The chips that were incubated in higher concentrations of Aβ42 standard exhibited higher overall resonant wavelength shift after the detection antibody was injected. The binding curves from Fig. 2a were fitted with a one-site specific binding model (Eq. 2) to obtain the extrapolated maximum binding signal, *Y*_*max*_. A calibration curve for Aβ42 was constructed by fitting a 5-parameter logistic model to the *Y*_*max*_ of each sample (Fig. 2b).

### Participant group demographic and clinical screening characteristics.

Amyloid plaque and neurofibrillary tangle densities were evaluated in the frontal, temporal, parietal, hippocampus, and entorhinal cortex of post-mortem participants using Thioflavine S, Campbell-Switzer, and Gallyas staining methods. Each area was graded using published CERAD templates^42^ and received a score of 0–3, with a combined score of 0–15 for all five areas. A higher score indicates the presence of more frequent plaques and tangles, while a lower score indicates none to sparse presence of plaques and tangles. The CSF samples screened for Aβ42 were grouped into low and high amyloid plaque/neurofibrillary tangle groups based on the median plaque/tangle scores for the samples being screened. The median amyloid plaque density score was 4, so a low amyloid plaque density score was defined as 4 or less, while a high amyloid plaque density score was defined as greater than 4. The median tangle density score was 7, so a low neurofibrillary tangle density score was defined as 7 or less and a high tangle density score was defined as greater than 7.

**Figure 3:**
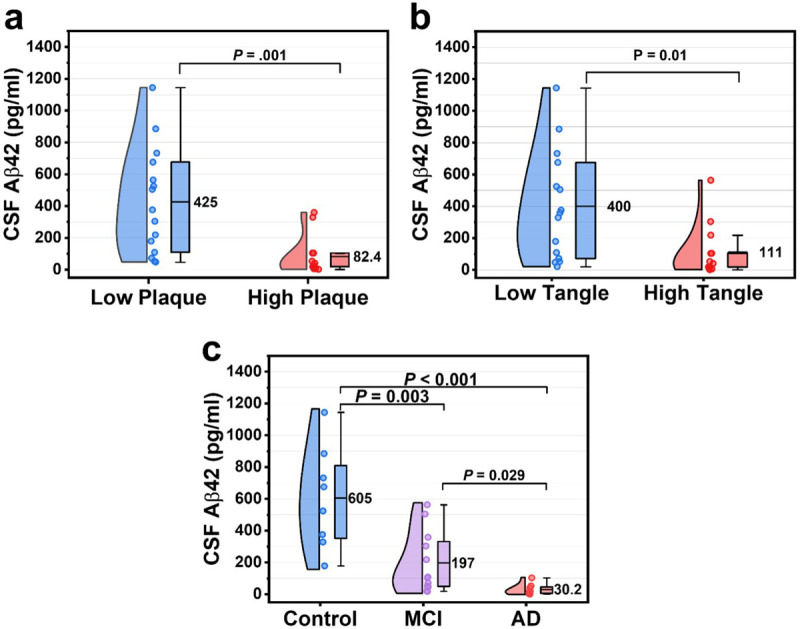
Measurement of CSF Aβ42 using FLOWER sandwich assay. (a) Aβ42 levels in CSF for low plaque vs. high plaque groups. Box charts show the mean, first, and third quartile. Whiskers show 1.5x IQR. (b) Aβ42 levels in CSF in participants grouped by their neurofibrillary tangle density score. (c) Aβ42 levels in CSF for clinicopathologically classified control, MCI, and AD groups.

Figure 3 shows the Aβ42 levels in post-mortem CSF measured using the FLOWER sandwich assay. When the CSF samples were grouped based on their total amyloid plaque density score, the mean (± SD) CSF Aβ42 levels in the low plaque group (425 ± 332 pg/mL) were 5-fold higher than the high plaque group (82.4 ± 120 pg/mL, *P* = 0.001) (Fig. 3a). When the same samples were grouped based on their total tangle density score, mean CSF Aβ42 levels in the low tangle group (400 ± 342 pg/mL) were significantly higher than those in the high tangle group (111 ± 163 pg/mL, *P* = 0.01). The presence of both amyloid plaques and neurofibrillary tangles is required for the neuropathological diagnosis of AD vs. other types of dementia. While neurofibrillary tangles are known to be neurotoxic, disrupt neuronal function, and lead to neuronal cell death^49^, the role of amyloid plaques in neurodegeneration is not as clear. Thus, there is still ongoing intense debate on whether it is the accumulation of Aβ or hyperphosphorylated tau that initiates AD, which one is more strongly associated with the progression of AD, and which should be targeted for therapeutic drugs^50,51^. *In-vivo* PET imaging has shown correlations between amyloid plaques, neurofibrillary tangles, and AD biomarkers in CSF and blood obtained in living participants. Similarly, we found that *in-vitro* measurement of post-mortem CSF Aβ42 with the FLOWER assay is associated with the neuropathology of AD.

Although there is currently no cure for AD, therapeutic drugs for reducing amyloid plaques^9,52^ and slowing the progression of dementia are an active, albeit controversial^53^, area of interest for AD research. For the early detection of AD, it would be valuable to be able to detect the disease in the earliest stages before the severe symptoms of dementia manifest. Thus, the same CSF samples were also grouped into 3 groups: control, MCI, and AD based on post-mortem neuropathology using CERAD-NP^42^ and NIA-R criteria^54^. In Fig. 3c, we observed significantly lower levels of CSF Aβ42 levels in the AD group (30.2 ± 34 pg/mL) compared to the MCI (197 ± 192 pg/mL), and control (605 ± 317 pg/mL) participant groups. The results of Fig. 3c show that the WGM biosensor was able to discriminate between control, MCI, and AD participants which provides initial support for its potential value in aiding early diagnosis.

**Figure 4:**
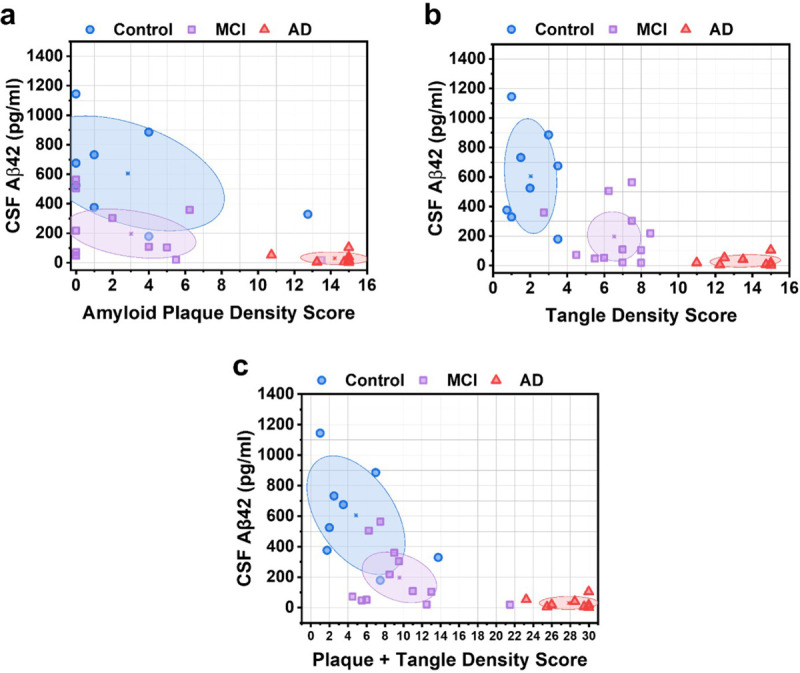
CSF Aβ42 vs. total plaque and total tangle density scores. (a) CSF Aβ42 vs. amyloid plaque density score. Ellipses depict the centroid (mean) of each group and the 95% confidence region. (b) CSF Aβ42 vs. neurofibrillary tangle density score. (c) CSF Aβ42 vs. combined plaque and tangle density score.

For each participant, the measured CSF Aβ42 level was evaluated against their respective total amyloid plaque and neurofibrillary tangle density score. The ellipses in Fig. 4 show the centroid (mean) of each group and the shaded area is the 95% confidence region. In Fig. 4a, Spearman correlation analysis revealed a moderately negative association between CSF Aβ42 and amyloid plaque density (ρ=−0.63, P < 0.001). These results are consistent with analysis of in vivo PET imaging of amyloid plaque deposition and CSF Aβ42 levels, which indicates that increased amyloid plaque load in the brain is associated with decreased CSF Aβ42 levels.^11^ In Fig. 4b, Spearman correlation analysis between CSF Aβ42 and neurofibrillary tangle density revealed a moderately negative correlation (ρ=−0.73, P < 0.001). Interestingly, we observed a distinct separation between control, MCI, and AD participants depending on their tangle density score in Fig. 4b. Compare this to Fig. 4a, where CSF Aβ42 levels are decreased for MCI subjects compared to healthy subjects, but with similar plaque density scores.

**Figure 5:**
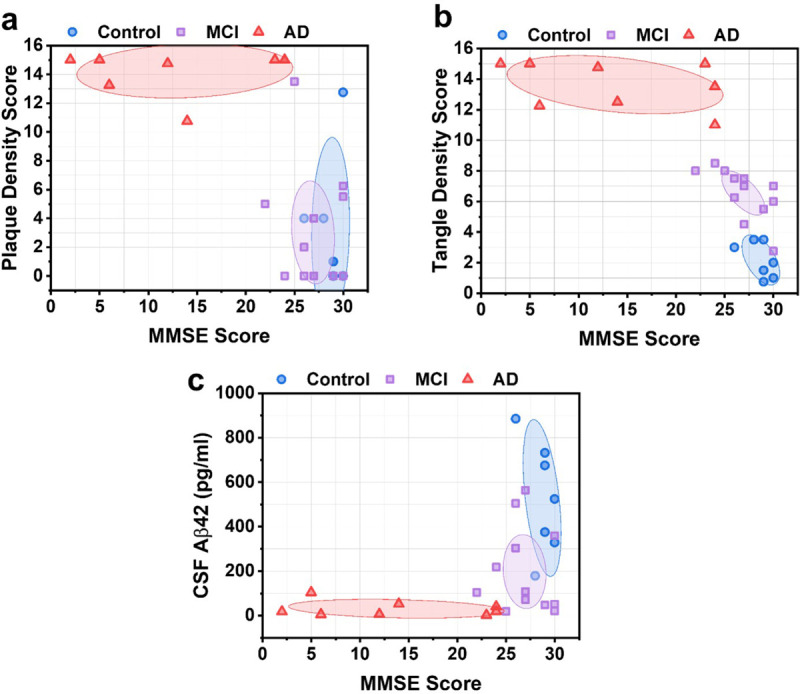
Plaque, tangle, and CSF Aβ42 associations with MMSE score. (a) Amyloid plaque density score vs. MMSE score. Ellipses depict the centroid (mean) of each group and the 95% confidence region. (b) Neurofibrillary tangle density score vs. MMSE score. (c) CSF Aβ42 vs. MMSE score.

We compared each subject’s plaque and tangle density scores vs. their MMSE scores, and the CSF Aβ42 measured by FLOWER vs. MMSE score. In Fig. 5, we observed a negative Spearman correlation for both plaque density vs. MMSE score (ρ=−0.59, *P* = 0.001) and tangle density vs. MMSE score (ρ=−0.86, *P* < 0.001), with distinct clustering between the control, MCI, and AD groups depending on tangle density score. There was a positive Spearman correlation between CSF Aβ42 levels and MMSE score (ρ = 0.52, *P* = 0.005).

**Figure 6:**
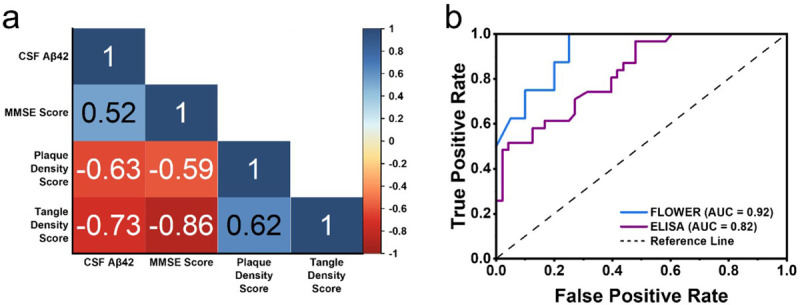
(a) Correlation matrix for CSF Aβ42. (b) ROC curve for CSF Aβ42 comparing FLOWER assay and ELISA.

Figure 6a presents the Spearman correlation matrix of CSF Aβ42 with relevant AD factors. Interestingly, we observed slightly stronger associations for CSF Aβ42 levels and MMSE scores with the tangle density score than the plaque density score. We also observed more clearly defined separation between MCI and control groups when looking at their tangle density scores compared to plaque density scores. These results suggest that tangle density may be a more sensitive measure than amyloid plaque density for this cohort. The diagnostic performance of the CSF Aβ42 FLOWER assay vs. an ultrasensitive human Aβ42 ELISA kit was compared through ROC curve analysis. Participants who were diagnosed with AD were categorized as the positive group (FLOWER n = 8, ELISA n = 31) while the control and MCI subjects were categorized as the negative group (FLOWER n = 19, ELISA n = 48). The CSF Aβ42 FLOWER assay displays better performance than previously reported ELISA^55^ (AUC = 0.82) in living participants and our own ELISA (AUC = 0.82) (Fig. S3) in post-mortem samples, and achieved an AUC = 0.92 (Fig. 6b). Additionally, this AUC was achieved using only Aβ42 as the biomarker rather than the Aβ42/Aβ40 ratio which reduces assay cost and complexity.

## CONCLUSION

We demonstrated for the first time the use of FLOWER for measuring Aβ42, an AD related biomarker, in clinicopathologically classified post-mortem human CSF. Our measurements showed that decreased CSF Aβ42 levels were associated with higher frequencies of amyloid plaque and neurofibrillary tangles in brain, the presence of which is required for the pathological diagnosis of AD. Additionally, CSF samples were sorted into control, MCI, and AD groups based on post-mortem neuropathology and clinical diagnosis, and FLOWER was able to differentiate healthy cognitively unimpaired participants from MCI and AD patients, an important step for early diagnosis. Using receiver operating characteristic analysis, FLOWER achieved higher diagnostic performance for CSF Aβ42 than the gold standard, ELISA (FLOWER AUC = 0.92 vs. ELISA AUC = 0.82), while also utilizing a single biomarker rather than the Aβ42/Aβ40 ratio. Our results demonstrate the potential capability of whispering gallery mode biosensors to aid early diagnosis of AD. Although our CSF was from post-mortem samples, the assay could also be applied to antemortem samples for longitudinal studies. In future studies, FLOWER will be extended to screening AD biomarkers in blood serum, which is an attractive alternative to CSF, due to the more complicated and invasive lumbar puncture procedure required to obtain repeated samples of CSF in living participants. Together, these findings suggest that FLOWER has potential to enhance fluid biomarker detection of AD, which may help to advance efforts in early diagnosis, as well as support potential applications in tracking disease progression and evaluating disease-modifying interventions.

## Figures and Tables

**Table 1:** Participant characterization for CSF samples.

Group	Sex (Male)	Age (Years)	PMI (hours)	MMSE Score	ApoE	Braak Stage
Control n = 8	0.625	82 ± 12	3.3 ± 0.99	28 ± 1.2	3/3, 3/3, 3/3, 2/3, 3/3, 3/3, 3/3, 3/3	1, 2, 3, 1,2, 3, 3, 1
MCI n = 11	0.45	91 ± 4.7	2.9 ± 0.69	26 ± 2.5	3/3, 3/3, 3/3, 3/3, 2/3, 3/3, 3/3, 3/3, 3/4, 2/3, 3/3	4, 4, 4, 4, 4, 4, 4, 4, 2, 4, 4
AD n = 8	0.50	71 ± 8.8	3.1 ± 0.67	13 ± 8.4	3/4, 3/4, 3/3, 3/4, 3/4, 3/3, 3/4, 3/3	5, 5, 6, 6, 5, 6, 6, 6

Values are the mean ± SD, PMI = post mortem interval between death and sample collection, MMSE = Mini Mental State Examination, ApoE = apolipoprotein E genotype

## Data Availability

The authors declare that the data supporting the findings of this study are available within the paper and its Supplementary Information files. Should any raw data files be needed in another format they are available from the corresponding author upon reasonable request.
